# Different HSP70 expression and cell survival during adaptive responses of 3T3 and transformed 3T3 cells to osmotic stress.

**DOI:** 10.1038/bjc.1993.92

**Published:** 1993-03

**Authors:** P. G. Petronini, R. Alfieri, E. De Angelis, C. Campanini, A. F. Borghetti, K. P. Wheeler

**Affiliations:** Istituto di Patologia Generale, Università degli Studi di Parma, Italy.

## Abstract

**Images:**


*Br. J. Cancer* (1993), **67**, 493–499 © Macmillan Press Ltd., 1993

### Different HSP70 expression and cell survival during adaptive responses of 3T3 and transformed 3T3 cells to osmotic stress

P.G. Petronini, R. Alfieri, E. De Angelis, C. Campanini, A.F. Borghetti & K.P. Wheeler

*Istituto di Patologia Generale, Università degli Studi di Parma, 43100 Parma, Italy and School of Biological Sciences, University of Sussex, Brighton BN1 9QG, UK.*

**Summary** Responses both to hyperosmotic stress and to heat shock were compared in 3T3 cells, spontaneously transformed cells (ST3T3) and simian virus 40-transformed cells (SV3T3). Cell adaptation to these stresses was measured in terms of surviving cell viability and plating efficiency, while their induced synthesis of stress proteins was monitored in terms of the presence of mRNA for HSP70, the pattern of polypeptides synthesised and the accumulation of HSP70 detectable by monoclonal antibodies. All three types of cells responded similarly to heat shock in their expression of HSP70 and showed no clear differences in ability to recover. In contrast, both ST3T3 and SV3T3 cells adapted more poorly and much more slowly to hyperosmotic stress (0.5 osM incubation) than did normal 3T3 cells. This different pattern of adaptation to hyperosmotic stress was parallelled by the cells' different expression of a stress protein that could not be distinguished from the heat-induced HSP70 by any of the methods listed above. In view of these findings it seems possible that hyperosmotic treatment might be useful in selectively affecting the survival of tumour cells.

Previous studies of cell metabolism and proliferation showed that transformed 3T3 cells adapted much more slowly than did 3T3 cells to hyperosmotic stress (Silvotti *et al.*, 1991). Several cell functions, such as amino acid transport activity, rate of protein synthesis and growth rate, returned to near normal values within 6 to 16 h in 3T3 cells but remained unadapted in transformed cells after 24 h. In parallel with these observations it was also shown that incubation in hypertonic medium for 6 h induced the synthesis of a 69 kDa protein in 3T3 cells, but not in transformed cells (Silvotti *et al.*, 1991). The obvious possibilities that this new stress protein could be a member of the family of 70 kDa heat shock proteins (HSP70) and might be involved in the adaptive response to hyperosmotic stress have therefore been investigated.

Responses both to hyperosmotic stress and to heat shock have been compared in 3T3 cells, spontaneously transformed 3T3 cells (ST3T3) and simian virus 40-transformed 3T3 cells (SV3T3). The induced synthesis of stress proteins by the cells under these conditions has been monitored in terms of the presence of mRNA for HSP70, the pattern of polypeptides synthesised, and the accumulation of HSP70 detectable by monoclonal antibodies.

### Materials and methods

### *Chemicals*

$\alpha[^{32}P]$dCTP, L-[4,5-$^{3}$H]Leucine, L-[$^{35}$S]methionine, and [$^{14}$C] methylated protein mixture (myosin, phosphorylase b, bovine serum albumin, ovalbumin, carbonic anhydrase, and lysozyme) and anti mouse actin monoclonal antibody were obtained from Amersham International plc, Amersham, Buckinghamshire, England. Pre-stained protein molecular weight standards were obtained from GIBCO BRL, Eggenstein, Germany. Monoclonal antibody directed against human inducible 72 kDa heat shock protein (C92F3A5) and monoclonal antibody directed against both human 72 kDa (inducible) and 73 kDa (constitutive) heat shock proteins (N27F3-4) were a generous gift of Dr William J. Welch (San Francisco). Plasmid pH2.3 containing the human HSP70 gene was kindly provided by Dr Richard I. Morimoto (Evanston) and was obtained through Dr Luisa Schiaffonati (Milan). Media, foetal calf serum (FCS), and antibiotics for culturing the cells were purchased from GIBCO, Grand Island, New York, NY, USA. Reagents of analytical grade were obtained from Sigma Chemicals Co., St. Louis, MO, USA, and from BDH Chemicals Ltd, Poole, Dorset, England. Reagents for electrophoresis and blotting analysis were obtained from BIO-RAD Laboratories, Richmond, California, USA.

### *Cell cultures*

Balb/c 3T3 cells (clone A 31) and simian virus 40 (SV40)-transformed Balb/c 3T3 cells (clone SV3T3) were obtained through Dr Salvatore Ruggieri (Florence) as described previously (Borghetti *et al.*, 1980). These cells were routinely kept in culture for up to 2 months and discarded; then fresh cultures were started again from frozen stocks. Spontaneously transformed 3T3 (ST3T3) cells were isolated in our laboratory on the basis of their loss of density control, serum dependence, ability to grow in soft agar medium, morphological modification and increased production of lactic acid.

The cells were maintained in Dulbecco's modified Eagle medium (D-MEM) containing penicillin (100 units ml$^{-1}$) and streptomycin (100 $\mu$g ml$^{-1}$) supplemented with 5% FCS for transformed cells and 10% FCS for 3T3 cells. Before an experiment was started the media were changed so that all cells, SV3T3, ST3T3 and 3T3, were incubated under identical conditions in the presence of 10% FCS. In all the cultures used the possible occurrence of mycoplasma contamination was tested at intervals with the use of a mycoplasma detection kit (Boehringer, Mannheim, Germany). All cultures were kept in an incubator at 37°C in a water-saturated atmosphere of 5% $CO_2$ in air, and were passaged twice a week. Measurements of the rate of protein synthesis were made on subcultures from cells plated on 9 cm$^2$ wells of disposable multiwell trays, whereas polypeptide and RNA profiles were determined in 27 or 81 cm$^2$ disposable plastic dishes. All subcultures were incubated for the desired period of time in 2, 6 or 18 ml, respectively, of complete growth medium. The seeding density was a quarter of the saturation density for 3T3 cells and approximately one twentieth for SV3T3 and ST3T3 cells. Under these culture conditions cells reached confluence within 4 days, the saturation density being 50,000–70,000 cells cm$^{-2}$ for 3T3 cells and about ten times higher for SV3T3 cells. For experiments cells were seeded at

Correspondence: K.P. Wheeler, School of Biological Sciences, University of Sussex, Brighton BN1 9QG, UK.
Received 29 June 1992; and in revised form 29 September 1992.

a density of about 10,000 cells $cm^{-2}$ and grown for only 2 days before the start of incubations. Thus all the cells were still growing and far from confluence. During experiments their densities were measured in terms of μg protein $cm^{-2}$ and the values for transformed cells were generally only 1.5 to 2 times those for the normal 3T3 cells.

*Culture media of altered hypertonicity*

Minimal essential medium used during experiments was brought to the desired $Na^+$ concentration by addition of 1.5 M NaCl. The correct final osmolarity of the modified medium was checked with a vapour pressure osmometer (Wescor). Normal medium contained 143 mM $Na^+$, 116 mM derived from NaCl, and the remainder from other components (sodium bicarbonate and sodium phosphate).

*Cell counting and determination of cell survival*

Cells were detached from the substratum by trypsinisation and appropriate dilutions of the suspension were counted in a Bürker haemocytometer, as described in detail elsewhere (Piedimonte et al., 1982). Cell survival was determined by cell viability and plating efficiency. After hyperosmotic treatment or heat shock, the cells were removed from the plates by trypsinisation, counted, and their viability determined by trypan blue exclusion (Hunt, 1987). The plating efficiency of viable cells was then determined by seeding them at an appropriate density in dishes containing 5 ml of culture medium. After 3 days of incubation the cells were detached from plates and counted.

*Protein labelling for polyacrylamide gel electrophoresis analysis (1D-Page)*

To label cell cultures for polyacrylamide gel electrophoresis (PAGE), methionine-free medium was supplemented with 1 μM unlabelled methionine and with 100 μCi $ml^{-1}$ [$^{35}$S] methionine (1115 Ci $mmol^{-1}$). At the end of the labelling period unlabelled methionine was added to reach a 1 mM final concentration. After 5 min of further incubation (chase period) the medium was discarded, the cell layer gently washed with 3 × 2 ml of cold phosphate buffer solution and then solubilised in buffer containing 10 mM NaCl, 3 mM $MgCl_2$, 10 mM Tris-HCl (pH 7.4), 0.1% sodium dodecyl sulphate (SDS), 0.1% Triton-X-100, 10 μg $ml^{-1}$ 4-amidophenyl-methanesulphonylfluoride, 0.5 μg $ml^{-1}$ leupeptin, 0.7 μg $ml^{-1}$ pepstatin and 0.5 mM EDTA. After sonication, samples corresponding to $3 \times 10^5$ c.p.m. were lyophilised and resuspended in sample buffer containing 62.5 mM Tris-HCl (pH 7.4), 2% SDS, 5% 2-mercaptoethanol, 0.002% bromophenol blue, and 20% glycerol. The samples were heated to 100°C for 2–3 min and then analysed by 1D-PAGE on slab gels using a standard apparatus (Bio-Rad). Equal amounts of radioactivity were loaded on the gel. Proteins were separated on 5–15% (w/v) linear gradient polyacrylamide gels (cross-linker = 1/38) in a discontinuous buffer system as described by Laemmli (1970), with a constant current of 35 mA for approximately 5 h. The gels were then fixed, dried, exposed to Kodak X-omat X-ray film for a week and developed.

*Two-dimensional protein analysis (2D-PAGE)*

When proteins were electrophoretically separated on two-dimensional gels the standard procedure first described by O'Farrel (1975) was followed with slight modifications. Aliquots of sonicated cell extracts (see previous paragraph) were lyophilised and resuspended in a solution containing 9.5 M urea, 5% 2-mercaptoethanol, 2% Nonidet P-40, 1.5% SDS, and 2% ampholites. Proteins, loaded on 1.5 mm-diameter gel rods, were focused in the first dimension with the use of 2% ampholites (Bio-Lyte ampholine 1.8% pH 5–7, 0.2% pH 3–10) for 14–15 h at 400 v, followed by 2 h at 800 v, constant voltage. Approximately $1.5 \times 10^6$ c.p.m. were loaded on each gel rod. Proteins were separated in the second dimension on 10% polyacrylamide gels in the presence of SDS. Two-dimensional analysis was performed in the Bio-Rad Protean II apparatus. Following electrophoresis, gels were dried and the autoradiograms were obtained as previously described for 1D-PAGE, after 3-weeks exposure.

*Peptide mapping*

One dimensional peptide mapping after limited proteolysis was performed as described by Cleveland et al. (1977) with minor modifications. The labelled protein samples were first separated on 5–15% linear gradient gels as described above. The gels were stained for 30 min in a solution containing 0.1% Coomassie blue, 50% methanol, and 10% acetic acid, and destained for less than an hour in a solution of 5% methanol and 10% acetic acid. The 70 kDa protein bands induced by heat shock or hyperosmotic treatment were individually cut out and equilibrated in digestion buffer containing 0.1% SDS, 0.125 M Tris-HCl (pH 6.8), 1 mM EDTA, and 0.0001% bromophenol blue. The gel slices were then applied to the sample wells of a second 5–15% SDS-gel, together with 200 ng of *Staphylococcus aureus* V8 protease (Sigma). Electrophoresis was carried out at 6 mA for 16 h. The gels were prepared for autoradiography as described above.

*Western blotting*

Protein content was determined by a dye-fixation method (BioRad) with bovine serum albumin as standard (Bradford, 1976). Immunoblotting analysis was performed essentially as described by Burnette (1981). Briefly, cell proteins separated by SDS-PAGE were electrophoretically transferred (30 V, overnight at 4°C) from the slab gel to a nitrocellulose sheet in a Trans Blot Cell (BIO-RAD) filled with cold blot buffer (25 mM Tris, 192 mM glycine, pH 8.3, 20% methanol). Non-specific protein binding was blocked by incubating sheets for an hour at room temperature (about 25°C) in blocking solution (20 mM Tris-HCl, 500 mM NaCl, pH 7.5, 3% gelatin). the saturated transfer membrane was washed twice (5 min per wash) with Tris Buffered Saline (TBS: 20 mM Tris-HCl, 500 mM NaCl, pH 7.5) containing 0.05% tween-20. The blot was then incubated for 4 h at room temperature with a 1:5000 dilution of the antibody in Antibody Buffer (AB: 20 mM Tris-HCl, 500 mM NaCl, pH 7.5, 0.05% tween-20, 1% gelatin). Blots were washed twice with TTBS (TBS containing 0.05% tween-20), incubated for 1 h at room temperature with a 1:3000 dilution in AB of goat anti-mouse Ig-G conjugated to alkaline phosphatase, then washed twice with TTBS and

**Table I** Cell viability and plating efficiency after heat shock or exposure to hypertonic medium

| Assay | Condition | *Per cent of control* 3T3 | ST3T3 | SV3T3 |
|---|---|---|---|---|
| Cell Viability | Hypertonic | 74 ± 17 (4) | 38 ± 5 (7) | 40 ± 4 (9) |
| | Heat shock | 54 ± 14 (9) | 68 ± 8 (7) | 71 ± 5 (6) |
| Plating Efficiency | Hypertonic | 77 ± 3 (4) | 11 ± 6 (8) | 9 ± 6 (12) |
| | Heat shock | 84 ± 15 (8) | 52 ± 6 (8) | 57 ± 22 (8) |

All cells were first grown for 3 days under 'normal' conditions in complete culture medium. Control cells were then incubated under the same conditions for a further 24 h. 'Heat shock cells' were incubated in the same medium, but at 44°C, for 0.5 h, followed by 23.5 h at 37°C. 'Hypertonic cells' were incubated for 24 h in complete growth medium plus about 0.1 M NaCl, i.è. in a 0.5 osM medium. All cells were then analysed for their viability and plating efficiency as described in the text. Mean values (± s.d.) from the number of independent measurements given in backets. Control values (cells 27 $cm^{-2}$) for cell visability ranged from $0.9 \times 10^6$ to $3.2 \times 10^6$ (3T3), $5.9 \times 10^6$ to $16.5 \times 10^6$ (ST3T3) and $8.6 \times 10^6$ to $13 \times 10^6$ (SV3T3). Control values (cells 27 $cm^{-2}$) for plating efficiency ranged from $1 \times 10^6$ to $3.2 \times 10^6$ (3T3), $1.7 \times 10^6$ to $9.7 \times 10^6$ (ST3T3) and $1.9 \times 10^6$ to $10.5 \times 10^6$ (SV3T3).

**Figure 1** Two-dimensional gel electrophoresis of L-[$^{35}$S]methionine-labelled proteins of 3T3 cells. 3T3 cells were incubated at 37°C for 6 h in control medium **a**, 0.5 OSM medium **b**, or incubated at 44°C in isotonic medium for 30 min and then at 37°C under recovery conditions for 6 h **c**. Cell proteins were labelled during 6 h of treatment with L-[$^{35}$S]methionine. Following a 5 min chase period, proteins were extracted from the cells separated by 2D-PAGE, and those labelled were visualised by autoradiography, as described in the text. Arrow indicates the HSP70 inducible form.

once with TBS. Detection of the antigen-antibody complex was carried out by incubating the sheet with colour development solution (15 mg of nitro blue tetrazolium, 0.7% N,N-dimethylformamide, 30 mg of 5-bromo-4-chloro-3-indolyl phosphate per 100 ml, 1 mM $MgCl_2$, and 100 mM $NaHCO_3$, pH 9.8). The development was stopped by immersing the sheet in distilled water for 10 min.

*Northern blotting*

Total RNA was extracted from cultured cells by the guanidium-caesium trifluoroacetate method (Okayama *et al.*, 1987) using a RNA extraction kit (Pharmacia). Ten μg RNA samples were fractionated by 1.2% agarose gel electrophoresis and transferred to nylon filters. The quality and quantity of RNA blotted on membranes was checked by UV absorption. Plasmid pH 2.3 containing the human HSP 70 gene (Hunt & Morimoto, 1985) was nick-translated (Amersham kit N.5000) with $\alpha$[$^{32}$P]dCTP (3000 Ci mmol$^{-1}$). Membranes were pretreated and hybridised in 50% formamide, 7% SDS, 0.25 M $NaHPO_4$, 0.25 M NaCl, and 1 mM EDTA. Afterwards, they were washed five times with 2 × SSC solution (1 × SSC is 0.15 M, NaCl 0.015 M sodium citrate, pH 7) containing 0.1% SDS at room temperature for 10 min, once

**Figure 2** Comparison by peptide mapping of HSP70 induced in 3T3 cells by heat shock or hyperosmotic stress. 3T3 cells were either incubated at 44°C in isotonic medium for 30 min and their proteins labelled for 16 h during recovery at 37°C (lane 2 and 4), or incubated at 37°C under hypertonic conditions for 16 h and their proteins labelled during the treatment (lane 1 and 3). The radioactive proteins were extracted and samples separated on SDS-PAGE. Radioactive bands were cut from the gels and re-electrophoresed in the presence (lane 1 and 2) or in the absence (lane 3 and 4) of *Staphylococcus aureus* V8 protease. Molecular weight standards are shown at the left.

**Figure 3** Comparison of HSP70 induced by heat shock or hyperosmolar stress. For heat shock, 3T3 cells were incubated in isotonic medium at 44°C for 30 min and then at 37°C for 6 h. For hyperosmotic stress, 3T3 cells were incubated in a 0.5 OSM medium for 6 h. Control cells were incubated in isotonic medium at 37°C for 6 h. Cell proteins were then extracted, separated by SDS-PAGE blotted on to nitrocellulose and detected with a monoclonal antibody directed against inducible HSP70, as described in the text. Key: C, control; Hy, hyperosmotic stress; Hs, heat shock; HSP70 I, inducible HSP70.

with 1 × SSC containing 0.1% SDS at 42°C for 10 min, and twice with 0.1 × SSC at 42°C for 10 min. The membranes were then exposed for 12–24 h with intensifying screens at − 80°C.

### Results

### *Cell survival after exposure to heat or hyperosmotic stress*

The relative abilities of the three types of cells (3T3, ST3T3 and SV3T3) to adapt both to hyperosmotic treatment and to heat shock are compared in Table I. Adaptation was assessed in terms of (a) cell viability and (b) cell plating efficiency. The 3T3 cells clearly adapted better to hypertonic conditions than did either of the transformed cells – considerably better in terms of viability and much better in terms of plating efficiency. In contrast, the comparative responses of 3T3 and the transformed cells to heat shock were both less pronounced and less consistent. Thus although plating efficiency did give the same pattern, but with much smaller and more variable differences, the cell viability assay showed that both types of transformed cells recovered from heat shock somewhat better than did the 3T3 cells. (Note that either form of stress might well interfere with cell membrane integrity in a way that renders the cell viability assay unreliable. The plating efficiency assay is a more reliable method of assessing the cells' condition).

**Figure 4** Accumulation of inducible HSP70 in cells during hypertonic incubation or recovery from heat shock. For heat shock, cells (3T3, ST3T3 and SV3T3) were incubated in isotonic medium at 44°C for 30 min and then at 37°C for recovery periods of 6 to 24 h. For hyperosmotic stress, cells were incubated in 0.5 OSM medium at 37°C for 6 to 24 h. Control cells were incubated in isotonic (0.3 OSM) medium throughout. Cell proteins were then extracted, separated by SDS-PAGE, blotted on to nitrocellulose and reacted with antibodies as described in the text. **a**, Detection with monoclonal antibody directed against inducible HSP70. **b**, Detection with monoclonal antibody directed against both constitutive and inducible HSP70. An anti-actin antibody was used as a control. Key: C, control; Hy, hyperosmotic stress; HS, heat shock; HSP70 C, constitutive HSP70; HSP70 I, inducible HSP70.

**Figure 5** The pattern of protein synthesis induced by hyperosmotic stress or heat shock in 3T3, ST3T3 and SV3T3 cells. **a**, For hyperosmotic stress the cells were incubated at 37°C in a 0.5 osm medium for 4, 8, 12, 16 or 24 h. For heat shock the cells were incubated in isotonic medium at 44°C for 30 min and then at 37°C for 4, 8 or 12 h of recovery. During the last 2 h of hyperosmotic treatment or recovery period the cells were labelled with [$^{35}$S]methionine. Following a 5 min chase period, proteins were extracted from the cells and separated by SDS-PAGE, and those labelled during the pulse were visualised by autoradiography, as described in the text. Key: lanes 1–9, 3T3 cells; lanes 10–18, ST3T3 cells; lanes 19–27, SV3T3 cells. **b**, Enlargement of zone containing inducible (I) and constitutive (C) isoforms of HSP70.

*Comparison of stress proteins induced by heat shock or hyperosmotic treatment*

Figure 1 shows an analysis by 2-dimensional gel electrophoresis of both the protein induced in 3T3 cells during hypertonic incubation and the HSP70 produced by these cells during their recovery from heat shock. Clearly both proteins migrated to the same position. Similarly, the patterns of peptides produced by partial proteolysis of the two stress proteins were indistinguishable (Figure 2). Finally, Figure 3 shows that the stress protein generated by exposure of the cells to hyperosmotic stress was recognised by a monoclonal antibody specifically directed against an inducible human HSP70. Taken together these findings indicate that hyperosmotic stress induces 3T3 cells to synthesise a protein that is highly homologous, if not identical, with a HSP70.

*Variation in the rate of accumulation of HSP70*

Two classes of monoclonal antibodies, one directed against the inducible form of HSP70 and another that recognises both the inducible and constitutive isoforms, were used to monitor the accumulation of these stress proteins during cell recovery from heat shock and cell adaptation to hyperosmotic incubation. The results (Figure 4) revealed a significant difference between the normal and transformed cells. In 3T3 cells heat shock and hypertonic treatment both induced an accumulation of HSP70 that was detectable after 6 h, reached a maximum around 16 h (particularly evident after heat shock) and persisted for at least 24 h. In transformed cells, however, there was a marked difference between the responses to heat shock and hyperosmotic stress. After heat shock the time course of accumulation of the inducible HSP70 in transformed cells parallelled that described above for 3T3 cells, whereas during hypertonic incubation the induced HSP70 was detectable only after 16 h. Thus the relative inability of the transformed cells to adapt to hyperosmotic stress after 6 h of treatment (Table I) was paralleled by their much slower accumulation of induced HSP70.

*Kinetics of incubation of pulse-labelled polypeptides*

In view of the results described above, the kinetics of induction of HSP70 were examined. Figure 5 shows the patterns of polypeptides synthesised during cell adaptation to hypertonic incubation and during cell recovery following heat shock.

After exposure of 3T3 cells to a shock for 30 min, synthesis of the inducible HSP70 was apparent after 4 h of recovery, but disappeared at later times. During hypertonic incubation of 3T3 cells synthesis of the inducible HSP70 was detectable after 8 h, reached a maximum around 12 h and became negligible at later times. (It should be noted that an increased synthesis of the constitutive HSP70 also occurred after 8 and 12 h of hyperosmotic incubation).

In both spontaneously and virally transformed 3T3 cells exposed to heat shock the kinetics of induction of the HSP70 during the recovery phase were similar to those observed in normal 3T3 cells. In contrast, during hyperosmotic incubation of the transformed cells the synthesis of the inducible isoform of HSP70 was evident only after much longer treatment (16 to 24 h, see Figure 5b). Again, some increased synthesis of the constitutive isoform was also apparent, but with kinetics similar to those observed in normal 3T3 cells.

*HSP70 gene transcription*

Northern blotting analysis was used to gain some insight into the transcriptional control of HSP70 mRNA in normal and transformed cells during responses to heat shock and hyperosmotic stress. As is evident from Figure 6, the human genomic probe used recognises two transcripts in these cells, one of about 2.7 kb and another of about 2.4 kb. These should correspond to the inducible and constitutive HSP70 transcript, respectively (cf. Colotta *et al.*, 1990), the two having over 70% homology (Schlesinger *et al.*, 1982).

After 3 h of recovery from heat shock the 2.7 kb transcript was markedly induced in all three cell types. There was also some increase in the amount of the constitutive 2.4 kb transcript under these conditions. In contrast, the pattern of response to hyperosmotic incubation was different in each type of cell. In 3T3 cells the 2.7 kb transcript was strongly induced after only 3 h of treatment and remained high after 7 h. In ST3T3 cells some increase in this transcript was apparent after 3 h and more after 7 h; but it had largely disappeared by 16 h. In SV3T3 cells induction occurred even later, with only a trace after 3 h but with gradually increasing induction up to at least 16 h. In all 3 types of cell hyperosmotic stress also caused some increase in the amount of the 2.4 kb transcript after 3 h and 7 h. After 16 h of treatment, however, there appeared to be less of this constitutive transcript in both types of transformed cells than in the controls.

**Discussion**

The results presented above show clearly that, contrary to earlier indications, the 69 kDa protein previously identified in 3T3 cells as an inducible 'osmotic stress protein' (Silvotti *et al.*, 1991) is in fact either highly homologous or identical to the inducible isoform of HSP70. The preliminary analysis by Western blotting (Silvotti *et al.*, 1991) was done after 4 h exposure to hyperosmotic conditions, a period based on ready detection of HSP70 during recovery from heat shock. It is now apparent that at least 6 h of hyperosmotic treat-

**Figure 6** Expression of HSP70 mRNA in 3T3 and transformed 3T3 cells. Cells were either incubated at 44°C in isotonic medium for 30 min and then at 37°C for recovery (HS), or incubated at 37°C in hypertonic medium (Hy), for the period indicated in figure. 'C' represents untreated control cells. At the time indicated total cellular RNA was extracted and analysed by Northern blotting, as described in the text. Key: 3T3 cells: lanes 1–4. ST3T3 cells: lanes 5–9. SV3T3 cells: lanes 10–14.

ment are required before enough of the protein has accumulated in 3T3 cells for detection by this method (Figure 4). Hence the initial conclusion that the 69 kD protein was unrelated immunologically to the inducible HSP70 is now known to be wrong, but explicable. Similarly, the earlier conclusion that hyperosmotic stress did not induce SV3T3 cells to produce the 69 kDa protein must be modified in the light of the present findings. Instead of an all or none response, the major difference between 3T3 and transformed 3T3 cells during hyperosmotic treatment was the length of time required before induction of synthesis of the stress protein was detectable. Transformed cells do eventually produce the stress protein in response to hyperosmotic incubation, but only after a much longer exposure to this stress than is required for untransformed cells.

Since the present findings indicate that heat shock and hyperosmotic treatment do induce the synthesis of the same HSP70 in these cells, it seems reasonable to assume that adaptation to either form of stress in both normal and transformed cells has common steps once the HSP70 genes are activated. If this is the case, there must be distinct pathways of HSP70 gene activation for the two types of stress. As far as hyperosmotic stress is concerned, it may be relevant that there were temporal differences between normal and transformed cells in the changes of concentrations of monovalent cations and some compatible osmolytes (Yancey *et al.*, 1982) during hyperosmotic incubation (Silvotti *et al.*, 1991). Hence it is possible that intracellular ion content, or compatible osmolyte concentration, may somehow control HSP70 gene activation. It is also worth noting that a decade ago Groudine and Weintraub (1982) reported that hyperosmotic NaCl shock of cultured cells induced DNAase I-hypersensitive sites, a trait shared by several genes undergoing transcription.

The question of whether or not HSP70 proteins actually mediate adaptation to hyperosmotic stress remains to be answered. The parallelism between HSP70 production and adaptation observed here is quite striking, but a causal relationship obviously does not necessarily follow. The parallel situation with regard to heat shock has remained unclear for years (cf. Burdon, 1986). For example, the recent findings of Bader *et al.* (1992), working with NIH 3T3 cells, appear to show conclusively that blocking the heat-induced expression of HSPs does not prevent either the development of thermal tolerance or the protection of protein synthesis after heating. On the other hand, Li *et al.*, (1992) have provided equally convincing evidence with gene transfer experiments that rat fibroblasts expressing a cloned human gene encoding an intact HSP70 become resistant to heat.

Regardless of the actual mechanism involved, however, the results presented above show that transformed 3T3 cells adapt poorly and much more slowly to hyperosmotic stress than do normal 3T3 cells. Hence the possibility of using hyperosmotic treatment, in place of or in addition to hyperthermia, might be considered as a means of selectively affecting the survival of tumour cells.

This investigation was supported by grants from MURST 60%, the MURST/British Council agreement, CNR, Rome; from AIRC, Milano; and the Associazione Chiara Tassoni, Parma, Italy.

### References

BADER, S.B., PRICE, B.D., MANNHEIM-RODMAN, L.A. & CALDERWOOD, S.K. (1992). Inhibition of heat shock gene expression does not block the development of thermotolerance. *J. Cell. Physiol.*, **151**, 56–62.

BURDON, R.H. (1986). Heat shock and the heat shock proteins. *Biochem. J.*, **240**, 313–324.

BORGHETTI, A.F., PIEDIMONTE, G., TRAMACERE, M., SEVERINI, A., GHIRINGHELLI, P. & GUIDOTTI, G.G. (1980). Cell density and amino acid transport in 3T3, SV3T3 and SV3T3 revertant cells. *J. Cell. Physiol.*, **105**, 39–49.

BRADFORD, M. (1976). A rapid and sensitive method for the quantitation of microgram quantities of protein utilizing the principle of protein-dye binding. *Anal. Biochem.*, **72**, 248–254.

BURNETTE, W.N. (1981). Western blotting: electrophorectic transfer of proteins from sodium dodecyl sulfate-polyacrylamide gels to unmodified nitrocellulose and radiographic detection with antibody and radioiodinated protein A. *Anal. Biochem.*, **112**, 195–203.

CLEVELAND, D.W., FISCHER, S.G., KIRSCHNER, M.W. & LAEMMLI, U.K. (1977). Peptide mapping by limited proteolysis in sodium dodecyl sulfate and analysis by gel electrophoresis. *J. Biol. Chem.*, **99**, 387–403.

COLOTTA, F., POLENTARUTTI, N., STAFFICO, M., FINCATO, G. & MANTOVANI, A. (1990). Heat shock induces the transcriptional activation of c-fos protooncogene. *Biochim. Biophys. Res. Comm.*, **168**, 1013–1019.

GROUDINE, M. & WEINTRAUB, H. (1982). Propagation of globin DNAaseI-hypersensitive sites in absence of factors required for induction: a possible mechanism for determination. *Cell*, **30**, 131–139.

HUNT, C. & MORIMOTO, R.I. (1985). Conserved features of eukaryotic hsp70 genes revealed by comparison with the nucleotide sequence of human hsp70. *Proc. Natl Acad. Sci. USA*, **82**, 6455–6459.

HUNT, S.V. (1987). Preparation of lymphocytes and accessory cells. In *Lymphocytes: A Practical Approach*. Klaus, G.G.B. (ed.) IRL Press, Ltd: Oxford, England, pp. 1–34.

LAEMMLI, U.K. (1970). Cleavage of structural proteins during assembly of the head of bacteriophage T4. *Nature*, **227**, 680–685.

LI, G.C., LI, L., LIU, R.Y., REHMAN, M. & LEE, W.M.F. (1992). Heat shock protein hsp70 protects cells from thermal stress even after deletion of its ATP-binding domain. *Proc. Natl Acad. Sci. USA*, **89**, 2036–2040.

O'FARREL, P.H. (1975). High resolution two-dimensional electrophoresis of proteins. *J. Biol. Chem.*, **250**, 4007–4021.

OKAYAMA, H., KAWAICHI, M., BROWNSTEIN, M., LEE, S., YOKOTA, T. & ARAI, K. (1987). High-efficiency cloning of full-length cDNA; construction and screening of cDNA expression libraries for mammalian cells. *Methods Enzymol.*, **154**, 3–28.

PIEDIMONTE, G., BORGHETTI, A.F. & GUIDOTTI, G.G. (1982). Effect of cell density on growth rate and amino acid transport in simian virus 40-transformed 3T3 cells. *Cancer Res.*, **42**, 4690– 4693.

SCHLESINGER, M.J., ALIPERTI, G. & KELLEY, P.M. (1982). The response of cells to heat shock. *Trends Biochem. Sci.*, **7**, 222–225.

SILVOTTI, L., PETRONINI, P.G., MAZZINI, A., PIEDIMONTE, G. & BORGHETTI, A.F. (1991). Differential adaptive response to hyperosmolarity of 3T3 and transformed SV3T3 cells. *Exp. Cell Res.*, **193**, 253–261.

YANCEY, P.H., CLARK, M.E., HAND, S.C., BOWLUS, R.D. & SOMERO, G. (1982). Living with water stress: evolution of osmolyte systems. *Science*, **217**, 1214–1222.


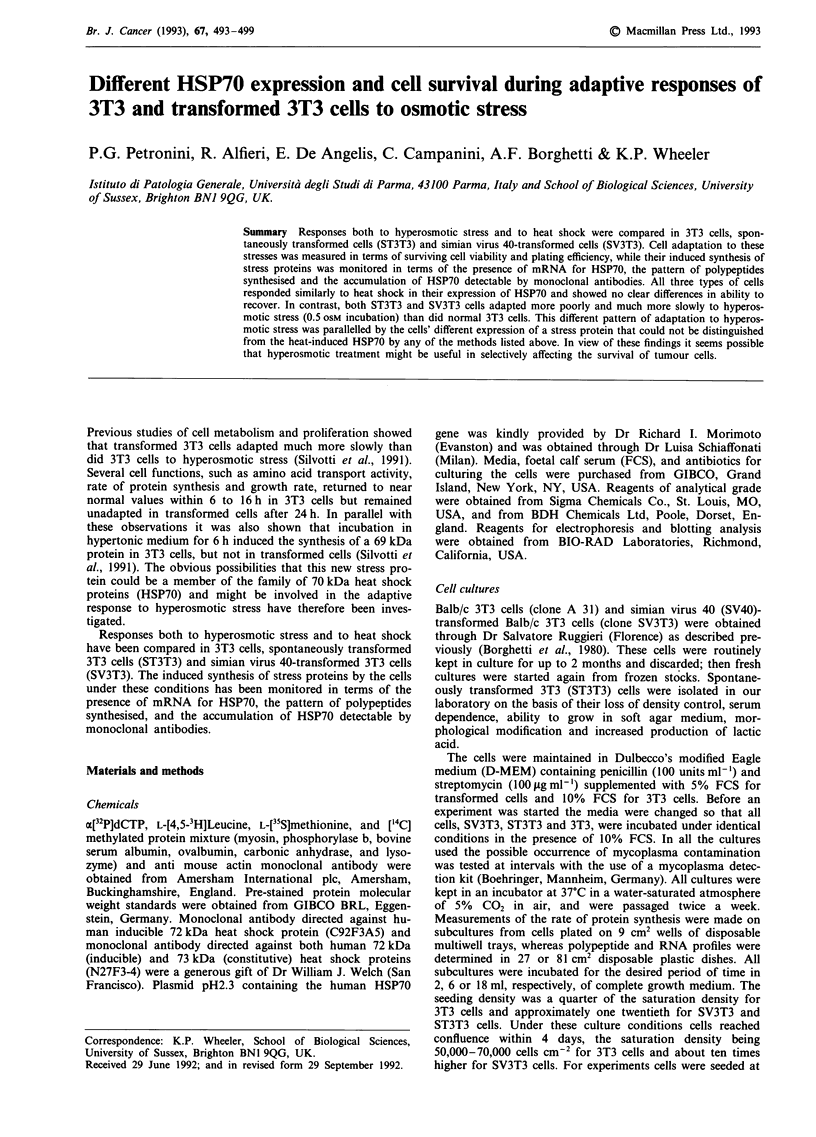

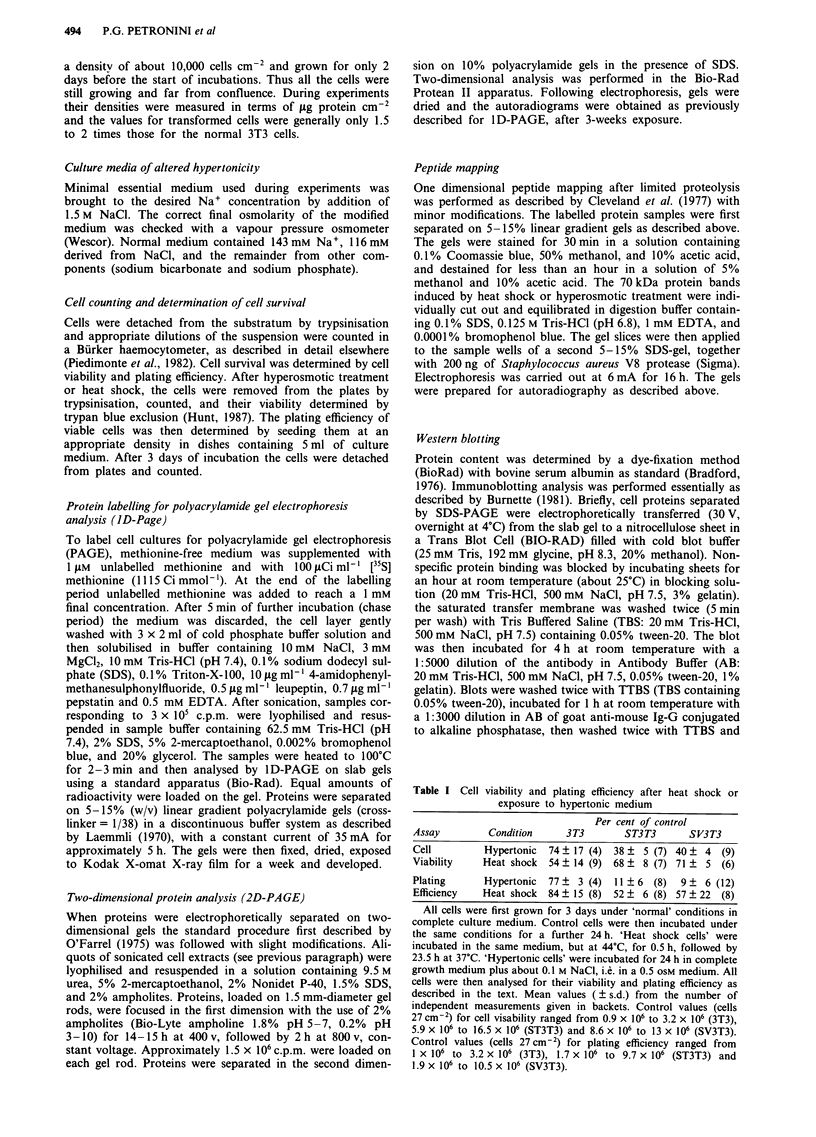

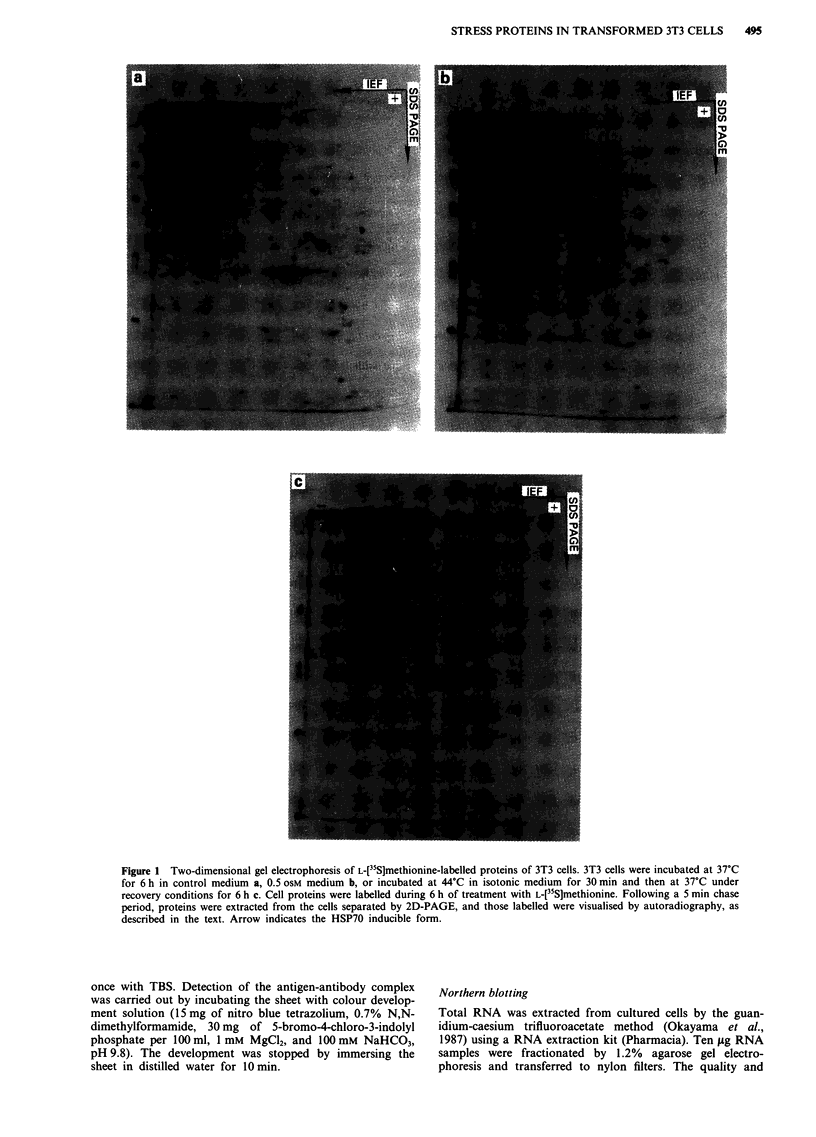

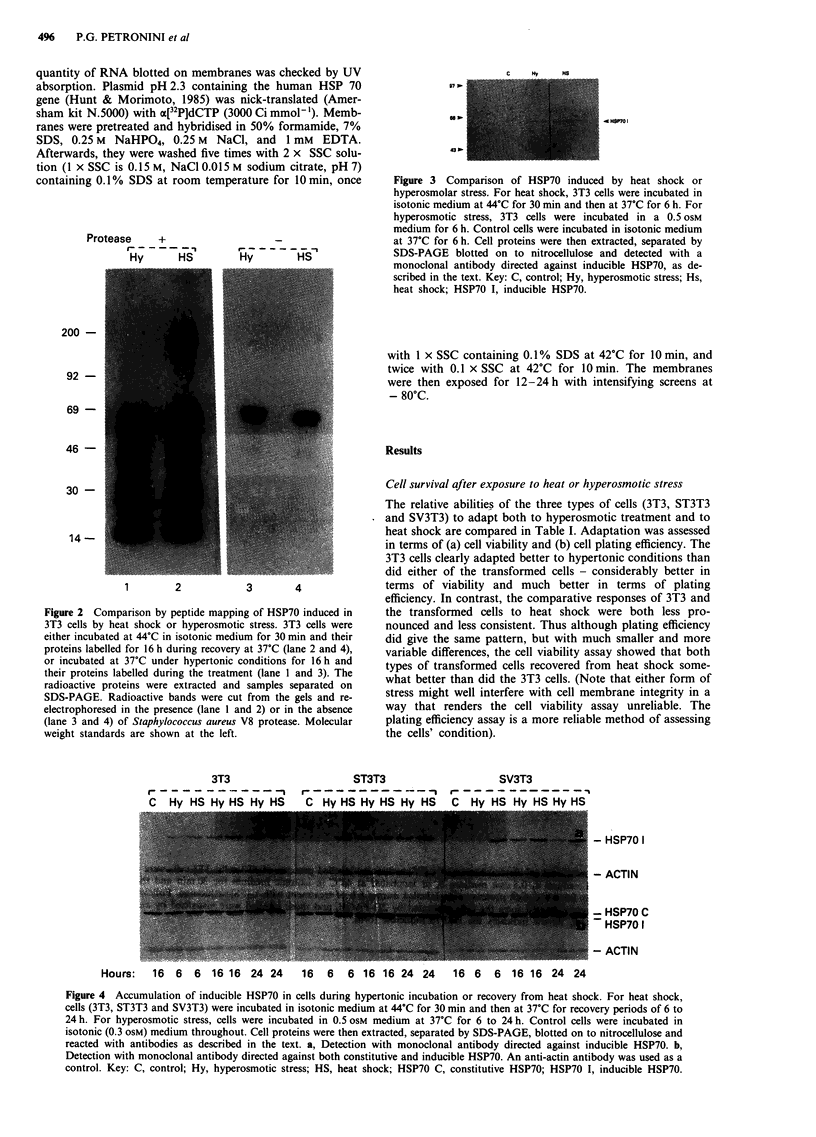

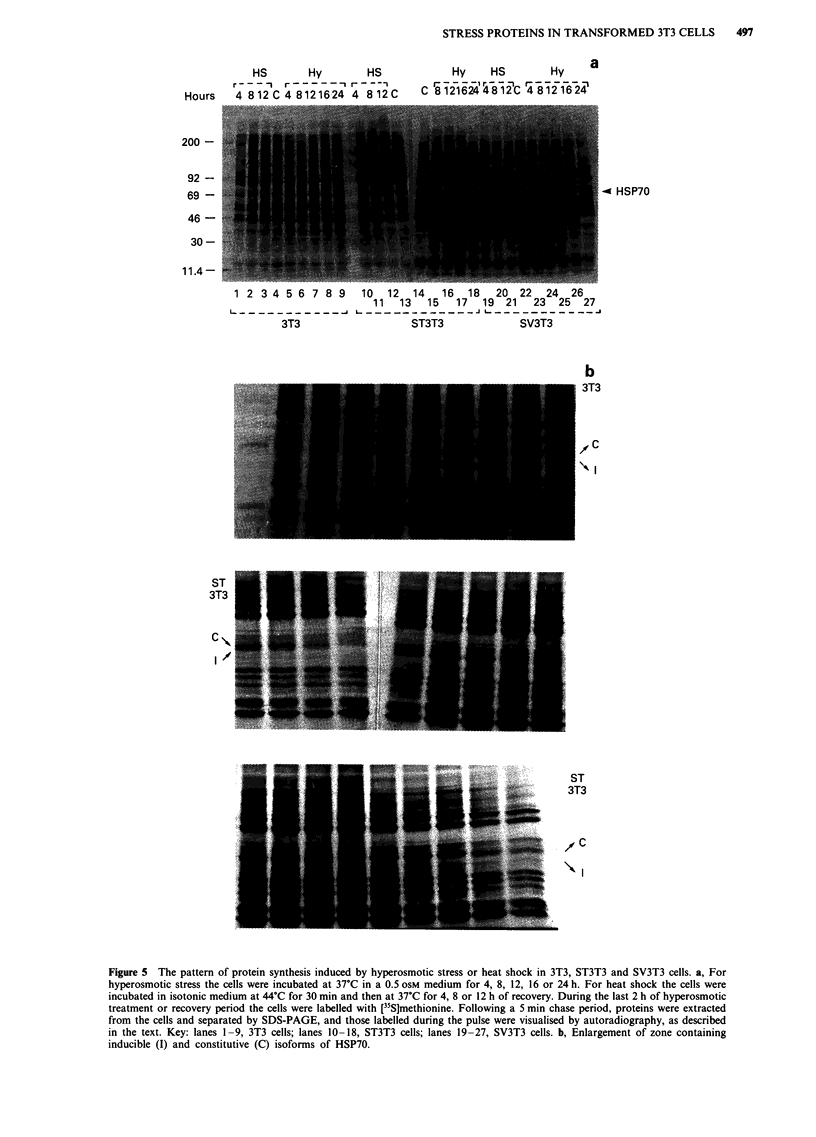

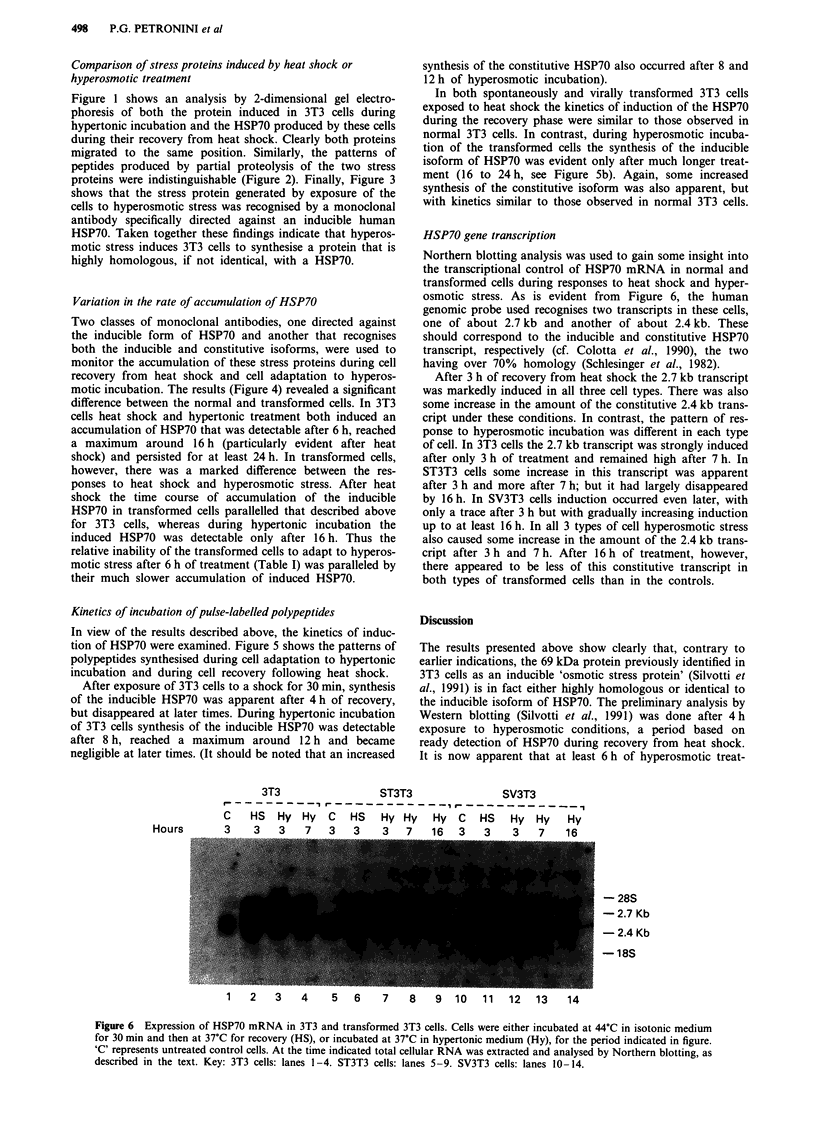

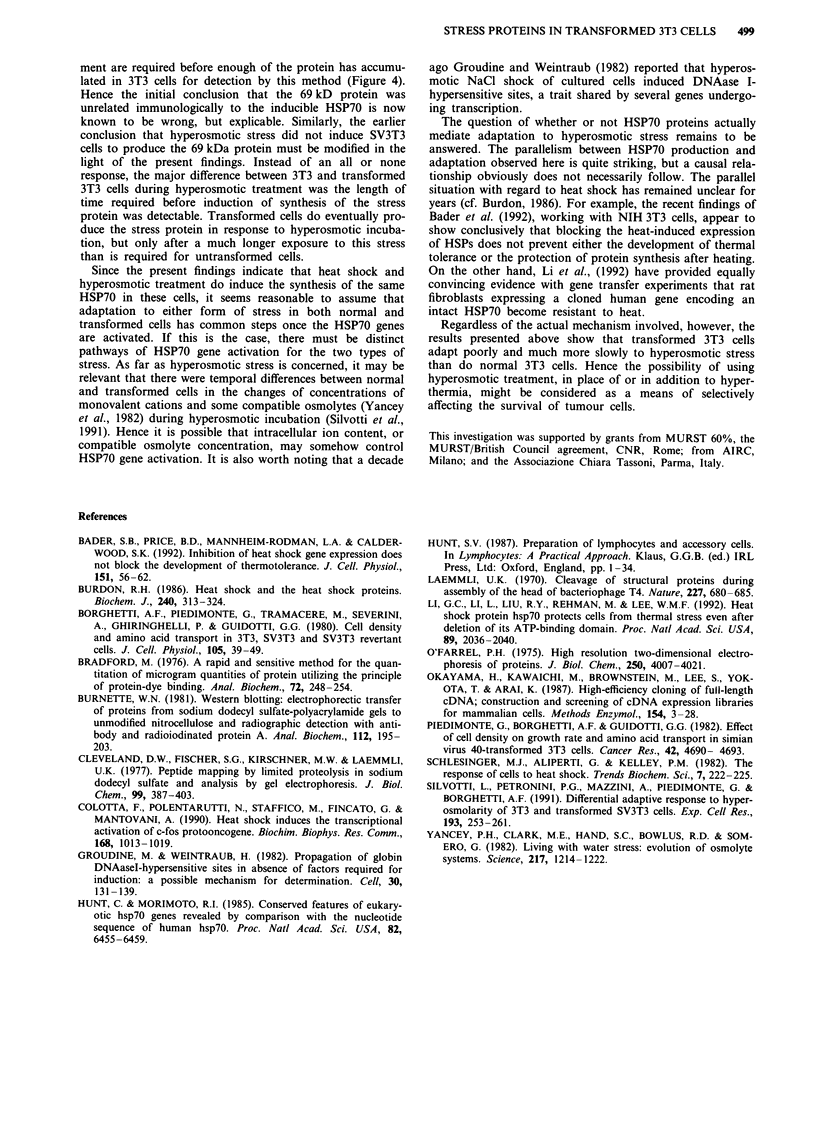


## References

[OCR_00681] Bader S. B., Price B. D., Mannheim-Rodman L. A., Calderwood S. K. (1992). Inhibition of heat shock gene expression does not block the development of thermotolerance.. J Cell Physiol.

[OCR_00689] Borghetti A. F., Piedimonte G., Tramacere M., Severini A., Ghiringhelli P., Guidotti G. G. (1980). Cell density and amino acid transport in 3T3, SV3T3, and SV3T3 revertant cells.. J Cell Physiol.

[OCR_00695] Bradford M. M. (1976). A rapid and sensitive method for the quantitation of microgram quantities of protein utilizing the principle of protein-dye binding.. Anal Biochem.

[OCR_00685] Burdon R. H. (1986). Heat shock and the heat shock proteins.. Biochem J.

[OCR_00700] Burnette W. N. (1981). "Western blotting": electrophoretic transfer of proteins from sodium dodecyl sulfate--polyacrylamide gels to unmodified nitrocellulose and radiographic detection with antibody and radioiodinated protein A.. Anal Biochem.

[OCR_00713] Colotta F., Polentarutti N., Staffico M., Fincato G., Mantovani A. (1990). Heat shock induces the transcriptional activation of c-fos protooncogene.. Biochem Biophys Res Commun.

[OCR_00719] Groudine M., Weintraub H. (1982). Propagation of globin DNAase I-hypersensitive sites in absence of factors required for induction: a possible mechanism for determination.. Cell.

[OCR_00725] Hunt C., Morimoto R. I. (1985). Conserved features of eukaryotic hsp70 genes revealed by comparison with the nucleotide sequence of human hsp70.. Proc Natl Acad Sci U S A.

[OCR_00736] Laemmli U. K. (1970). Cleavage of structural proteins during the assembly of the head of bacteriophage T4.. Nature.

[OCR_00739] Li G. C., Li L., Liu R. Y., Rehman M., Lee W. M. (1992). Heat shock protein hsp70 protects cells from thermal stress even after deletion of its ATP-binding domain.. Proc Natl Acad Sci U S A.

[OCR_00745] O'Farrell P. H. (1975). High resolution two-dimensional electrophoresis of proteins.. J Biol Chem.

[OCR_00751] Okayama H., Kawaichi M., Brownstein M., Lee F., Yokota T., Arai K. (1987). High-efficiency cloning of full-length cDNA; construction and screening of cDNA expression libraries for mammalian cells.. Methods Enzymol.

[OCR_00755] Piedimonte G., Borghetti A. F., Guidotti G. G. (1982). Effect of cell density on growth rate and amino acid transport in simian virus 40-transformed 3T3 cells.. Cancer Res.

[OCR_00763] Silvotti L., Petronini P. G., Mazzini A., Piedimonte G., Borghetti A. F. (1991). Differential adaptive response to hyperosmolarity of 3T3 and transformed SV3T3 cells.. Exp Cell Res.

[OCR_00771] Yancey P. H., Clark M. E., Hand S. C., Bowlus R. D., Somero G. N. (1982). Living with water stress: evolution of osmolyte systems.. Science.

